# OsCYP21-4, a novel Golgi-resident cyclophilin, increases oxidative stress tolerance in rice

**DOI:** 10.3389/fpls.2015.00797

**Published:** 2015-10-01

**Authors:** Sang S. Lee, Hyun J. Park, Won Y. Jung, Areum Lee, Dae H. Yoon, Young N. You, Hyun-Soon Kim, Beom-Gi Kim, Jun C. Ahn, Hye S. Cho

**Affiliations:** ^1^Sustainable Bioresource Research Center, Korea Research Institute of Bioscience and BiotechnologyDaejeon, South Korea; ^2^Molecular Breeding Division, National Academy of Agricultural Science, Rural Development of AgricultureJeonju, South Korea; ^3^Department of Pharmacology, College of Medicine, Seonam UniversityNamwon, South Korea

**Keywords:** cyclophilin, golgi-resident protein, oxidative stress, peroxidase activity, PPIase, salinity tolerance

## Abstract

OsCYP21-4 is a rice cyclophilin protein that binds to cyclosporine A, an immunosuppressant drug. CYP21-4s in Arabidopsis and rice were previously shown to function as mitochondrial cyclophilins, as determined by TargetP analysis. In the current study, we found that OsCYP21-4-GFP localized to the Golgi, rather than mitochondria, in *Nicotiana benthamiana* leaves, which was confirmed based on its co-localization with *cis* Golgi α-ManI-mCherry protein. *OsCYP21-4* transcript levels increased in response to treatments with various abiotic stresses and the phytohormone abscisic acid, revealing its stress-responsiveness. CYP21-4 homologs do not possess key peptidyl prolyl cis/trans isomerase (PPIase) activity/cyclosporine A (CsA) binding residues, and recombinant OsCYP21-4 protein did not convert the synthetic substrate Suc-AAPF-pNA via *cis- trans-* isomerization *in vitro*. In addition, transgenic plants overexpressing *OsCYP21-4* exhibited increased tolerance to salinity and hydrogen peroxide treatment, along with increased peroxidase activity. These results demonstrate that OsCYP21-4 is a novel Golgi-localized cyclophilin that plays a role in oxidative stress tolerance, possibly by regulating peroxidase activity.

## Introduction

The Golgi is a highly dynamic organelle that serves as the major site for post-translational protein modification and synthesis of various polysaccharides and glycolipids destined for the cell wall and plasma membrane, respectively (Lerouxel et al., 2006; Nilsson et al., 2009). The Golgi also plays a defining role in the processing and sorting of transport (cargo) proteins, lipids, and complex carbohydrates to various destinations within most eukaryotic cells (Matheson et al., 2006; Nanjo et al., 2006).

Unlike in animal cells, the Golgi apparatus in plant cells is located close to the nucleus in a rather stationary state; the Golgi takes the form of numerous individual Golgi stacks, which are mostly regarded as functional features required for the synthesis and trafficking of complex carbohydrates to the cell wall and transport of proteins to organelles (Radhamony and Theg, 2006). Furthermore, plant Golgi stacks do not disassemble at any stage during mitosis, whereas mammalian Golgi stacks remain intact and increase somewhat in number throughout the cell cycle (Nebenführ et al., 2000; Faso et al., 2009; Ito et al., 2014). Plant cells contain between several and hundreds of distinct Golgi stacks (cisternae; six on average) and the functional subdivision of Golgi stacks into cis-, medial-, and trans-cisternae is based on enzyme activity (Dupree and Sherrier, 1998).

Nevertheless, despite numerous studies over the past 30 years, no consensus amino acid sequence that serves as a Golgi retention signal has been identified, but the transmembrane domain and the cytosolic tail appear to be involved in this process (Saint-Jore-Dupas et al., 2004). Numerous Golgi-resident proteins have been identified in human and mouse (1183), whereas only approximately 400 plant Golgi proteins have been experimentally verified (Parsons et al., 2012). Since plant Golgi proteins do not possess obvious target signals that help proteins localize to other subcellular compartments, the Golgi-resident prediction computational programs are less than adequate for determining their localization (Sprenger et al., 2006). Recently, Chou et al. developed a novel Golgi-prediction server, GolgiP, which predicts both transmembrane- and non-transmembrane-associated Golgi-resident proteins in plants (Chou et al., 2010). GolgiP was used to predict Golgi proteins in 18 fully sequenced plant genomes based on their functional domains, revealing that similar percentages of Golgi proteins are found among lower to higher plant species. GolgiP currently supplies multiple models for predicting plant Golgi proteins.

A growing body of evidence indicates that the Golgi apparatus participates in stress signaling sensing, although little is known about this process in plants. The Golgi apparatus is involved in oxidative stress-mediated pathogenesis (Hu et al., 2007; Fan et al., 2008), apoptosis (Hicks and Machamer, 2005), and endoplasmic reticulum (ER) stress (Xu et al., 2010) in mammalian cells. Some studies elucidated the relationship between the Golgi apparatus and signal transduction under oxidative stress (Zhou et al., 2007; Braga et al., 2008). Maturation of complex N-glycan is required for plant adaptation to salinity stress (Kang et al., 2008; Von Schaewen et al., 2008; Zhang et al., 2009), heat stress (Shiraya et al., 2015), and pathogen immunity (Häweker et al., 2010). However, although our knowledge of the importance of the Golgi apparatus under oxidative stress is advancing, the molecular mechanisms underlying this process are mostly unknown. Further investigation is essential for elucidating these underlying mechanisms.

Cyclophilns (CYPs), the target of the immunosuppressive drug CsA, belong to the peptidyl prolyl *cis/trans* isomerase superfamily and play central roles in various biological processes in living cells, including splicesome assembly (Horowitz et al., 2002; Mesa et al., 2008), RNA processing (Gullerova et al., 2006), protein trafficking (Freskgård et al., 1992; Ferreira et al., 1996), miRNA activity (Smith et al., 2009), complex assembly and stabilization (Iki et al., 2012), signal transduction (Brazin et al., 2002), cell division (Faure et al., 1998), and detoxification of reactive oxygen species (ROS) (Hong et al., 2002). Arabidopsis CYPs have been functionally well-characterized compared to other plant CYPs, playing roles in assembly and maintenance of PSII supercomplex (Fu et al., 2007), effector activation (Coaker et al., 2005), organogenesis (Li et al., 2007), transcription and pre-mRNA processing (Leverson and Ness, 1998), plastid cysteine biosynthesis (Dominguez-Solis et al., 2008), cellular redox homeostasis (Kopriva, 2013; Park et al., 2013b), and phytochrome and cryptochrome signaling (Kang et al., 2008; Trupkin et al., 2012; Ma et al., 2013). By contrast, in monocot rice, only a few cyclophilins have been characterized (Ruan et al., 2011; Kim et al., 2012; Kang et al., 2013). In a previous study, we analyzed stress-responsive CYPs in rice (Ahn et al., 2010) and characterized the Os CYPs involved in environmental stress defense (Kim et al., 2012; Park et al., 2013a; Seok et al., 2014; Lee et al., 2015). Nevertheless, much work on CYPs remains to be conducted, and there have been no previous reports on the functional analysis of Golgi-localized CYPs in different plants.

This study is the first to attempt the functional characterization of Golgi-localized CYP and the results may serve as a starting point for further studies concerning its role within the Golgi apparatus under cellular stress conditions.

## Materials and methods

### Bioinformatics prediction

The *OsCYP21-4* sequence was used as a query to search for OsCYP21-4 homologs from the NCBI database through BLAST analysis. The amino acid sequences from OsCYP21-4 and its homologs were aligned using ClustalW2 and GeneDoc2.7. The phylogenetic tree of CYP21-4 homologs was constructed using the neighbor-joining method in Molecular Evolutionary Genetics Analysis (MEGA; version 5). The accession numbers are as follows: OsCYP21-4, NP_001059626.1 (*Oryza sativa*); XP_003563133.1 (*Brachypodium distachyon*); BAJ94163.1 (*Hordeum vulgare*); NP_001146433.1 (*Zea mays*); AtCYP21-4, NP_187319.1 (*Arabidopsis thaliana*); XP_002882485.1 (*Arabidopsis lyrata*); XP_010464248.1 (*Camelina sativa*); XP_009147140.1 (*Brassica rapa*); CDY05284.1 (*Brassica napus*); XP_002277818.1 (*Vitis vinifera*); and XP_004246609.1 (*Solanum lycopersicum*). The OsCYP21-4 sequence was analyzed using WoLF PSORT (http://wolfpsort.org/), PSORT (http://psort.ims.u-tokyo.ac.jp/), TargetP 1.1 (http://www.cbs.dtu.dk/services/TargetP/), Predotar (http://urgi.versailles.inra.fr/predotar/predotar.html), MitoProtII (http://ihg.gsf.de/ihg/mitoprot.html), SignalP 4.1 (http://www.cbs.dtu.dk/services/SignalP/), GolgiP (http://csbl1.bmb.uga.edu/GolgiP/), and TMHMM Server v. 2.0 (http://www.cbs.dtu.dk/services/TMHMM/) programs.

### Plant materials, growth conditions, and stress treatments

Sterilized rice (*Oryza sativa* L. cv Dong Jin) seeds were embedded in 1/2MS medium and grown at 28°C for 1–2 weeks under a 12 h light/12 h dark cycle with 100 μE m^−2^s^−1^ light intensity, and several stresses treatments were performed as described previously (Lee et al., 2015). The seedlings were desiccated for drought stress treatment or treated with 100 μM ABA, 200 mM NaCl, 10 mM H_2_O_2_, and 10 μM MV and harvested at 0, 1, 3, 6, 12, and 24 h. Heat stress involved treatment at 42°C, followed by harvesting at 0, 0.1, 0.5, 1, 2, 3, and 4 h. Three experiments were performed per treatment, with at least three replicated measurements for each parameter assayed.

### Gene expression analysis

Total RNA was extracted from plants grown under normal or stress conditions using RNAiso Plus (TaKaRa, Tokyo, Japan). Total RNA treated with RNase-free DNase I (Fermentas, Burlington, Canada) was used for cDNA synthesis (RevertAid First-strand cDNA Synthesis Kit; Fermentas). Quantitative reverse transcription PCR (qRT-PCR) was performed in a CFX Connect™ Real-Time PCR Detection System (Bio-Rad, Hercules, CA, USA) using SYBR Premix Ex-Taq (TaKaRa), according to the manufacturer's instructions. Relative expression levels are presented after normalization with *OsACT1* expression levels. All RT-PCR experiments were performed in at least three biological replicates, each with three technical repeats, under the same conditions.

### Expression and purification of OsCYP21-4-His-tagged protein

Expression and purification of recombinant OsCYP21-4 were carried out using the Novagen pET28a vector according to the supplier's protocols (EMD Millipore, Darmstadt, Germany). *OsCYP21-4* was cloned into pET28a and sequenced. The *OsCYP21-4* construct was transformed into *Escherichia coli* BL21 (DE3) for expression of His-tagged OsCYP21-4, and recombinant protein was purified on nickel-NTA agarose columns. Finally, the concentration and purity of OsCYP21-4-His protein were determined using the Bradford assay (Bio-Rad) and SDS-PAGE analysis.

### Protease-coupled assay for PPIase activity

The PPIase activity of recombinant OsCYP21-4 was measured *in vitro* against a synthetic tetrapeptide with the composition N-succinyl-Ala-Ala-Pro-Phe-NA (Suc-AAPF-pNA; Sigma-Aldrich, St. Louis, USA) in a chymotrypsin-coupled assay (Fischer et al., 1984) with some modifications. A 6 mM Suc-AAPF-pNA substrate stock was prepared in trifluoroethanol containing 0.47 M LiCl. Assay blanks (1 mL total) contained 60 μL of chymotrypsin (10 mg/mL) and 20 μL of substrate stock in assay buffer (50 mM HEPES and 100 mM NaCl, pH 8.0). PPIase assays were identical to the blank assays, except that they included purified OsCYP21-4-His protein. Chymotrypsin and OsCYP21-4-His protein were mixed with assay buffer and transferred to a quartz cuvette. The substrate was then introduced into the cuvette and mixed. The absorbance of the solution at 390 nm was recorded immediately after mixing and monitored at 10°C for 300 s in a Shimadzu UV-2450 spectrophotometer (Shimadzu, Kyoto, Japan) with a thermostatically controlled cuvette holder. All assays were carried out in triplicate.

### Subcellular localization of OsCYP21-4 proteins

The subcellular localization of OsCYP21-4 was determined by creating fluorescent fusion proteins. *OsCYP21-4* and *OsCYP21-4* deletion fragments were inserted into binary vector pCAMBIA1302 containing the CaMV 35S promoter and GFP gene. To obtain *OsCYP21-4* and *OsCYP21-4* deletion fragments (OsCYP21-4TM, 1-57 amino acids; OsCYP21-4ΔTM, 58-235 amino acids) containing coding sequence without the stop codon, *OsCYP21-4* cDNAs were ligated into the Nde1/Spe1 sites upstream of the N-terminal end of the GFP (Supplementary Table S1). The constructs were transiently expressed in *N. benthamiana* leaves using agro-infiltration. Two days after infiltration, the leaves were examined by fluorescence or confocal microscopy (Park et al., 2013a). The OsCYP21-4TM-GFP construct was co-expressed with the RFP-labeled endoplasmic reticulum (ER) marker, BiP. Brefeldin A (BFA) was used to block secretion of OsCYP21-4-GFP to the Golgi apparatus. OsCYP21-4-GFP was transiently expressed in *N. benthamiana* treated with 50 μM BFA for 3 h.

### Generation and stress treatment of *OsCYP21-4* overexpressing plants

The full-length cDNA sequence of *OsCYP21-4* was cloned into pCAMBIA1300 under the control of the 35S promoter (Supplementary Table S1). Rice was transformed with the construct by *Agrobacterium*-mediated transformation (Hiei et al., 1994). Genomic DNA was isolated from the leaves of wild-type (WT) and transgenic plants. To identify positive transgenic plants, one primer was designed to bind to the promoter region and another was designed to bind to the *OsCYP21-4* cDNA region. WT plants were used as a control. The expression level of the *OsCYP21-4* transgene was determined by RT-PCR. Actin was used as a reference for normalization. For various stress treatments, transgenic and WT rice seeds were germinated on 1/2MS medium plates with (for transgenic lines) or without (for WT) hygromycin. After 3 days, the rice seedlings were transferred onto 1/2MS medium containing 150 mM NaCl or 5 μM ABA and grown for 5 days. The roots of 3-day-old rice seedlings, which were selected using the method described above (for salt treatment), were incubated in a solution of H_2_O_2_ and NaCl for 3 and 5 days, respectively. The fresh weight, and root or shoot length of each seedlings were measured in triple independent experiments.

### H_2_O_2_ detection

Plant leaves were excised and immersed in a solution of 1 mg/mL 3′,3′-diamino benzidine (DAB) in Tris-HCl buffer (pH 6.5) containing 0.01% Triton X-100. After vacuum-infiltration for 60 min, the samples were incubated at room temperature for 20 h in the dark. To remove chlorophyll, the leaves were bleached by boiling in ethanol for 20 min. Brown spots indicated the presence of H_2_O_2_ *in situ* (Thordal-Christensen et al., 1997). Two independent experiments were carried out.

### Peroxidase and catalase activity

To determine peroxidase (POD, EC 1.11.1.7), ascorbate peroxidase (APX, EC 1.11.1.11) and catalase (CAT, EC 1.11.1.6) activities, 1 g of frozen power from leaf samples was homogenized in ice-cold 100 mM potassium phosphate pH 6.0 (POD), 50 mM pH7.5 sodium phosphate buffer (APX), or 50 mM pH7.0 potassium phosphate buffer (CAT). The homogenate was centrifuged at 12,000 g for 20 min at 4°C, and the supernatant was used for enzyme activity determinations. POD, APX, and CAT activities were analyzed by monitoring the increase in absorbance at 420, 290, and 240 nm, respectively. POD and APX activities were assayed according to the method described by Kwak et al. using pyrogallol and ascorbate as substrates, respectively (Kwak et al., 1995). CAT activity was determined by monitoring the consumption of H_2_O_2_ (Aebi, 1984). All measurements of activity were performed in triplicate.

### Statistical analysis

Statistical differences between treatments on different samples were analyzed following the Student's *t*-test using Excel. Differences were considered significant at a probability level of *p* ≤ Diff, 0.1, 0.05, or 0.01.

## Results

### OsCYP21-4 is a CYP protein that is conserved in different plant species

**Figure 1:**
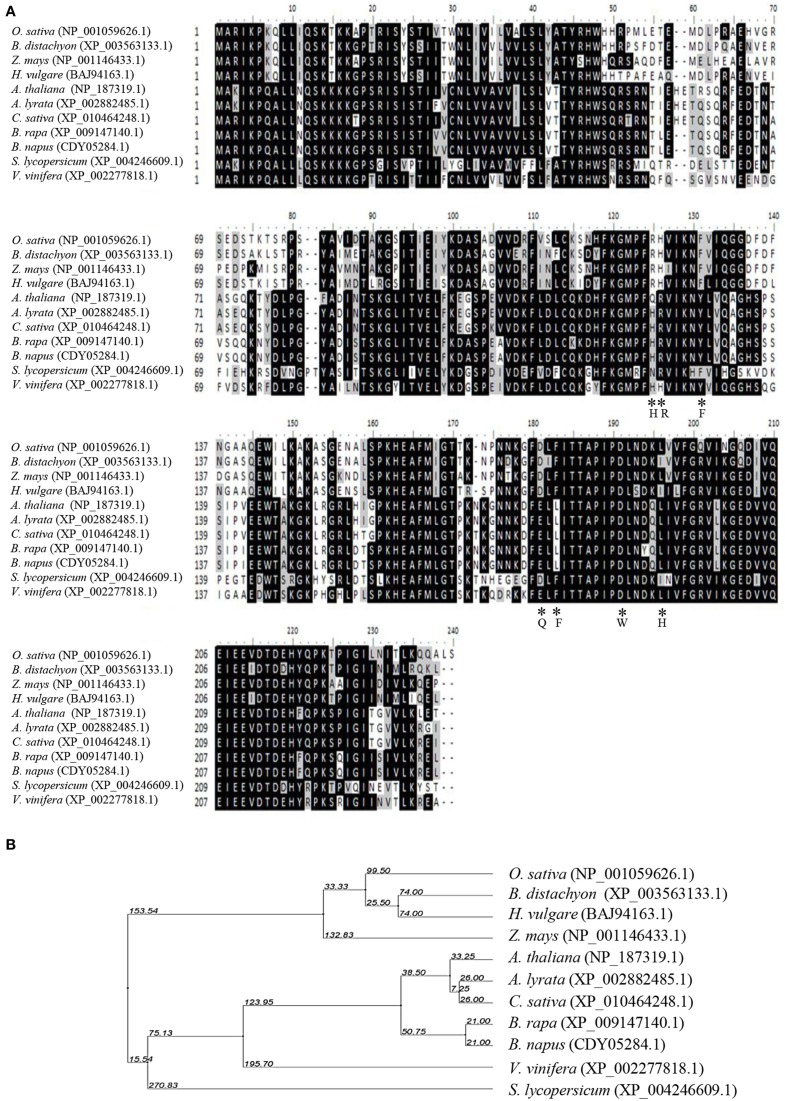
**Multiple sequence alignment and phylogenetic relationship between OsCYP21-4 and CYP21-4 homologs from various plants. (A)** Sequence comparison of OsCYP21-4 protein with selected CYP21-4 homologs from various plant species. The amino acids necessary for PPIase activity/CsA binding and are marked by asterisks. The degree of background shading indicates amino acid identity and similarity (*black: identity* >50%, *gray: similarity* > 50%). **(B)** Phylogenetic distance between OsCYP21-4 and other homologs.

OsCYP21-4 (Os07g29390) is a rice cyclophilin protein containing 235 amino acids with a molecular mass of 26.4 kDa. It contains a single CYP domain (amino acids 80–230). By searching NCBI (http://www.ncbi.nlm.nih.gov/BLAST/) using the OsCYP21-4 sequence as a query, we identified OsCYP21-4 homologs in three monocot and seven dicot plants (Figure 1A). Multiple amino acid sequence alignments showed that OsCYP21-4 shares high similarity with its homologs from monocots (84% similarity with *B. distachyon*, 81% with *H. vulgare*, and 81% with *Z. mays*) and less conserved sequence homology with CYP21-4 homologs from dicots (76% similarity with *V. vinifera*, 70% with Arabidopsis, 69% with *C. sativa*, and *Brassica*, and 67% with *S. lycopersicum*). Moreover, the results of phylogenetic tree analysis based on the full-length sequences of these homologs are good in agreement with the evolutionary relationships among these species (Figure 1B): OsCYP21-4 has a closer evolutionary relationship with CYP21-4 homologs from monocots than from dicots.

### OsCYP21-4 is localized to the golgi apparatus and BFA inhibits its localization

**Figure 2:**
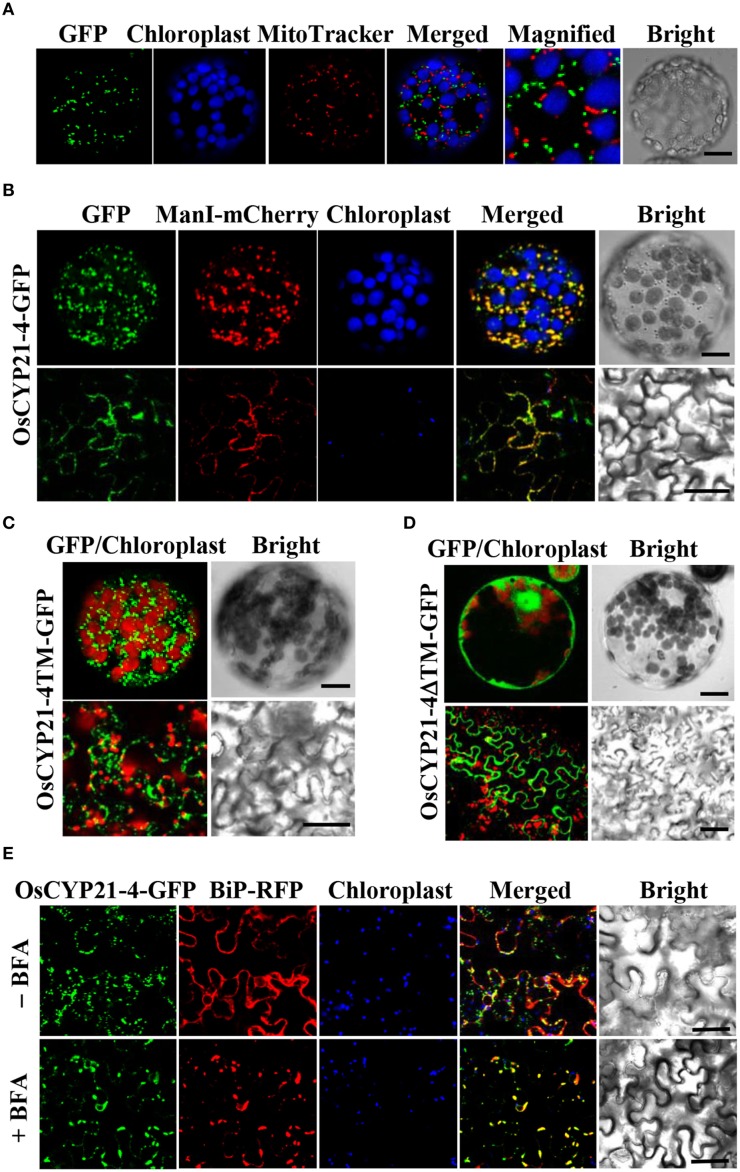
**Localization of OsCYP21-4-GFP in ***N. benthamiana***. (A)** OsCYP21-4-GFP did not co-localize with the mitochondrial marker, MitoTracker. **(B)** OsCYP21-4-GFP co-localized with the Golgi Apparatus marker α-ManI-mCherry. **(C,D)** Subcellular localizations of OsCYP21-4TM-GFP and OsCYP21-4ΔTM-GFP proteins. **(E)** Co-localization of OsCYP21-4-GFP with the ER marker, BiP-RFP under treatment with or without 50 μM BFA for 3 h. Bars = 20 μm.

Previous studies revealed that two CYP21-4s from Arabidopsis and rice are mitochondrial cyclophilins, as predicted using the TargetP program (He et al., 2004; Ahn et al., 2010). In the current study, we analyzed OsCYP21-4 using WoLF PSORT, MitoProtII, and Predotar as well as TargetP. The results of these predictions are summarized in Table 1. All of these programs except PSORT predicted that OsCYP21-4 is also localized to the mitochondria. To examine the intracellular localization of this protein, we cloned *OsCYP21-4* into plant expression vector pCAMBIA1302 between the 35S promoter and *GFP* gene (Supplementary Figure S1A), and transiently expressed the OsCYP21-4-GFP fusion protein in *N. benthamiana* leaves, followed by confocal laser scanning microscopy (CLSM) to observe its localization. OsCYP21-4-GFP was not clearly localized to the mitochondria, instead showing inconsistent localization patterns using the mitochondria marker MitoTracker (Figure 2A). We also found that GFP fused to OsCYP21-4 did not co-localize with the peroxisomal marker, RFP-SKL (three amino acids fused to red fluorescent protein) or with RFP-BiP ER-Tracker (Supplementary Figures S1B,C). Interestingly, a few punctuate OsCYP21-4-GFP signals were closely linked to or merged with the peroxisomal marker, and numerous punctuate signals were merged or closely linked to RFP-BiP (Supplementary Figures S1B,C: closed white arrowheads). Therefore, we cannot rule out ER-mediated localization of OsCYP21-4 in the cell.

**Table 1:** **Prediction of OsCYP21-4 subcellular localization by website programs**.

**WoLFPSORT**		**PSORT**		**TargetP**	**MitoProtII**	**Predotar**		**GolgiP**
Mito	8	PM	0.790	mTP (0.754)	Y (0.775)	Mito	(0.67)	Golgi (0.917)
Chlo	3	Chlo	0.399	47 residues		Else	(0.33)	Accuracy (34.2%)
Cyto	1	Golgi	0.300					
Pero	1	ER						

*For WoLF PSORT and PSORT predictions, the four most favorable localizations are reported with corresponding scores. For TargetP prediction, the scores of transit peptide presence and transit peptide length are given. For MitoProtII, Predotar and GolgiP predictions, the scores are given. cTP, chloroplast transit peptide; Mito, mitochondria; Chlo, chloroplast; Cyto, cytoplasm; Pero, peroxisome; mTP, mitochondria transit peptide; PM, plasma membrane. GolgiP prediction indicates the accuracy of prediction*.

Prediction by another program, GolgiP, which predicts Golgi-localized proteins in plants, supported the possibility that OsCYP21-4-GFP is localized to the Golgi, although the accuracy of the prediction is not high (Table 1). To verify that the fluorescent signals were indeed coming from the Golgi, we employed α-Mannosidase I fused to mCherry fluorescent protein (α-ManI-mCherry) (Nelson et al., 2007), which labels *cis* Golgi (Saint-Jore-Dupas et al., 2006). When OsCYP21-4 fused to GFP (OsCYP21-4-GFP) was co-expressed with α-ManI-mCherry, OsCYP21-4-GFP clearly co-localized with the α-ManI-mCherry fluorescence (Figure 2B). As determined by SignalP, a signal peptide predictor, OsCYP21-4 lacks a signal peptide. On the other hand, a traditional transmembrane topology predictor, TMHMM, predicted that an N-terminal transmembrane (TM) segment of OsCYP21-4 protein is a signal peptide (Supplementary Figure S1D). To analyze the signal peptide of Golgi-localized OsCYP21-4, we generated two OsCYP21-4 deletion constructs based on the results of TMHMM prediction. One construct contained the N-terminal TM segment (1-57 amino acid residues) of OsCYP21-4 fused to GFP (OsCYP21-4TM-GFP), and the other contained TM deleted-OsCYP21-4 fused to GFP (OsCYP21-4ΔTM-GFP) (Supplementary Figure S1E). OsCYP21-4TM-GFP localized to the Golgi, indicating that the N-terminal region of OsCYP21-4 is critical for its localization. By contrast, the OsCYP21-4ΔTM-GFP produced fluorescence only in the cytosol (Figures 2C,D).

Since OsCYP21-4 appears to be a Golgi-resident protein, trafficking of OsCYP21-4 should be inhibited by BFA, an inhibitor of the Golgi apparatus. To determine whether OsCYP21-4 trafficking to the Golgi is blocked by BFA, epidermal cells infiltrated with a mixture of *Agrobacterium* suspension harboring the OsCYP21-4-GFP construct and the silencing suppressor p19 were examined after 2 days of infiltration under a confocal microscope. Solutions containing infiltration buffer (as a negative control) or 50 μg/mL BFA in infiltration buffer were injected into leaves to reveal the Golgi localization of the fusion protein. The leaves were visualized 3 h after BFA injection using confocal microscopy (Figure 2E). Treatment with BFA resulted in enhanced intracellular GFP fluorescence in a pattern resembling the ER network, which was merged with the signal from red fluorescent protein fused with chaperone binding protein (BiP) (Kim et al., 2001), implying that BFA treatment prevented further translocation of OsCYP21-4 from the ER to the Golgi or caused the protein to relocate from the Golgi to the ER.

### Gene expression analysis of *OsCYP21-4*

**Figure 3:**
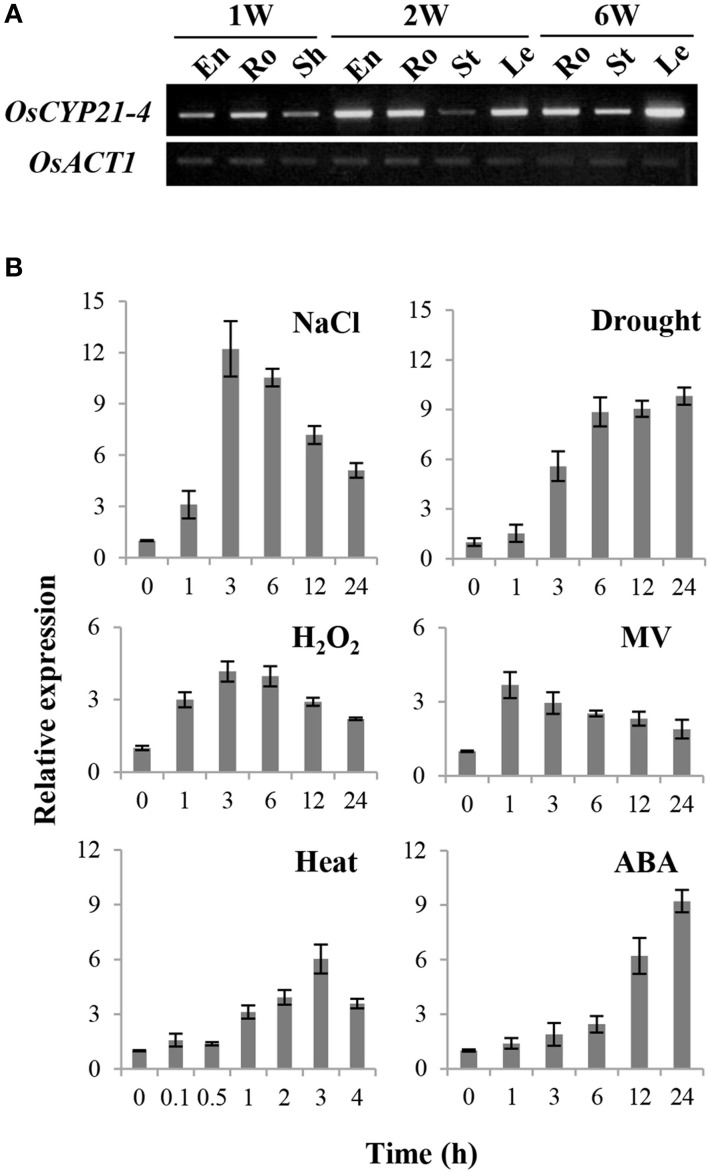
**Expression of ***OsCYP21-4*** in rice. (A)** Expression levels of *OsCYP21-4* in different tissues and stages by semi-quantitative RT-PCR analysis. 1 W, 1-week-old; 2 W, 2-week-old; 6 W, 6-week-old; En, endosperm; Ro, root; Sh, sheath; St, stem; Le, leaf; *OsACT1*, rice actin1. *OsACT1*, rice *actin1. OsACT1* was used as an internal control for mRNA quantification. **(B)** Expression levels of *OsCYP21-4* in 2-week-old rice seedlings in response to salt (200 mM), drought (desiccation), H_2_O_2_ (10 mM), MV (10 μM), heat (42°C), and ABA (100 μM) treatment. Relative expression levels of *OsCYP21-4* (revealed by qRT-PCR) were normalized to that of *OsACT1*. MV, methyl viologen; H_2_O_2_, hydrogen peroxide; ABA, abscisic acid.

To characterize the expression pattern of *OsCYP21-4* in rice, we analyzed the transcript levels of this gene at different developmental stages and in different tissues by semi-quantitative RT-PCR. *OsCYP21-4* was expressed differentially in various tissues at different stages during plant development. In 1-week-old seedlings, *OsCYP21-4* was expressed at higher levels in the roots than in the endosperm and sheath tissues. Two-week-old plants exhibited ubiquitous expression in the endosperm, roots, and leaves, except in stem tissue. On the other hand, in 6-week-old mature plants, *OsCYP21-4* was more highly expressed in leaves than in other tissues (Figure 3A).

To investigate the effects of abiotic stress on *OsCYP21-4* expression, we examined the expression patterns of this gene by qRT-PCR in rice seedlings subjected to salt, drought, H_2_O_2_, MV, heat, and ABA treatment. As shown in Figure 3B, *OsCYP21-4* expression increased 4–12-fold under various abiotic stress conditions. The expression of *OsCYP21-4* increased approximately 10-fold under high salinity, drought, and ABA stress conditions. Interestingly, the expression of *OsCYP21-4* was rapidly induced by MV, H_2_O_2_, high salinity and heat treatment within 1–3 h and gradually decreased thereafter. However, *OsCYP21-4* expression under drought and ABA treatment continued to increase until 24 h of treatment (Figure 3B). The up-regulated expression patterns of *OsCYP21-4* suggest that it may be involved in responses to such stresses.

### OsCYP21-4 does not have ppiase activity *in vitro*

**Figure 4:**
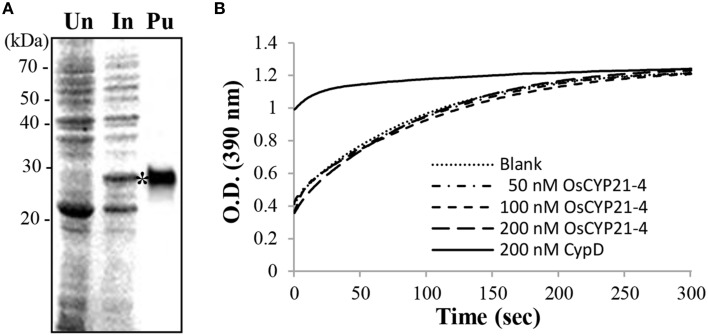
**Recombinant OsCYP21-4 lacks PPIase activity ***in vitro***. (A)** Recombinant OsCYP21-4 expression was induced with IPTG in *E. coli* and purified on nickel-NTA agarose columns. Samples were separated by 12% SDS-PAGE and stained with Coomassie blue. Un/In: Total protein un-induced/induced with isopropyl β-D-1-thiogalactopyranoside in *E. coli;* Pu: Purified OsCYP21-4 protein (star). **(B)** Traces showing isomerization of the Suc-AAPF-pNA substrate in the absence of OsCYP21-4 (Blank; …) and in the presence of 50 nM (50 nM OsCYP21-4;– -), 100 nM (100 nM OsCTP21-4;– -), and 200 nM (200 nM OsCTY21-4; _ _) recombinant OsCYP21-4 protein. CypD (200 nM CypD;) as a positive control. A representative recording from one of three independent experiments is shown.

The structure of OsCYP21-4 resembles that of other members of the CYP family in rice, and it contains a single CYP domain (80–230 amino acids). However, multiple amino acid sequence alignments revealed that the seven core amino acids necessary for CsA binding and PPIase activity, as determined for human CyPA (Zydowsky et al., 1992), are not conserved in CYP21-4 homologs (Figure 1A). In particular, OsCYP21-4 and its homologs from monocots contain only two conserved amino acid residues among the seven key amino acid residues, suggesting that these proteins lack PPIase activity. To determine whether OsCYP21-4 has PPIase activity, we expressed recombinant OsCYP21-4-His in *E. coli* and purified OsCYP21-4-His using nickel-affinity purification (Figure 4A). We measured the PPIase activity of OsCYP21-4, which is rate-limited by *cis*-*trans* isomerization of the Ala-Pro peptide bond of the synthetic peptide succinyl-Ala-Ala-Pro-Phe-p-nitroanilide, using a chymotrypsin-coupled PPIase assay. Kinetic data were obtained in the presence of increasing amounts of OsCYP21-4-His. The isomerization of the peptide substrate was not accelerated in the presence of 50, 100, or 200 nM OsCYP21-4-His, showing O.D. values identical to that of the blank, a negative control. Recombinant HsCypD protein (Giorgio et al., 2010) was used as a positive control (Figure 4B). These results suggest that OsCYP21-4 is not an active PPIase and that it may function as a PPIase-independent protein (like a chaperone) in the Golgi.

### Overexpression of *OsCYP21-4* increases salt tolerance and peroxidase activity in transgenic rice

**Figure 5:**
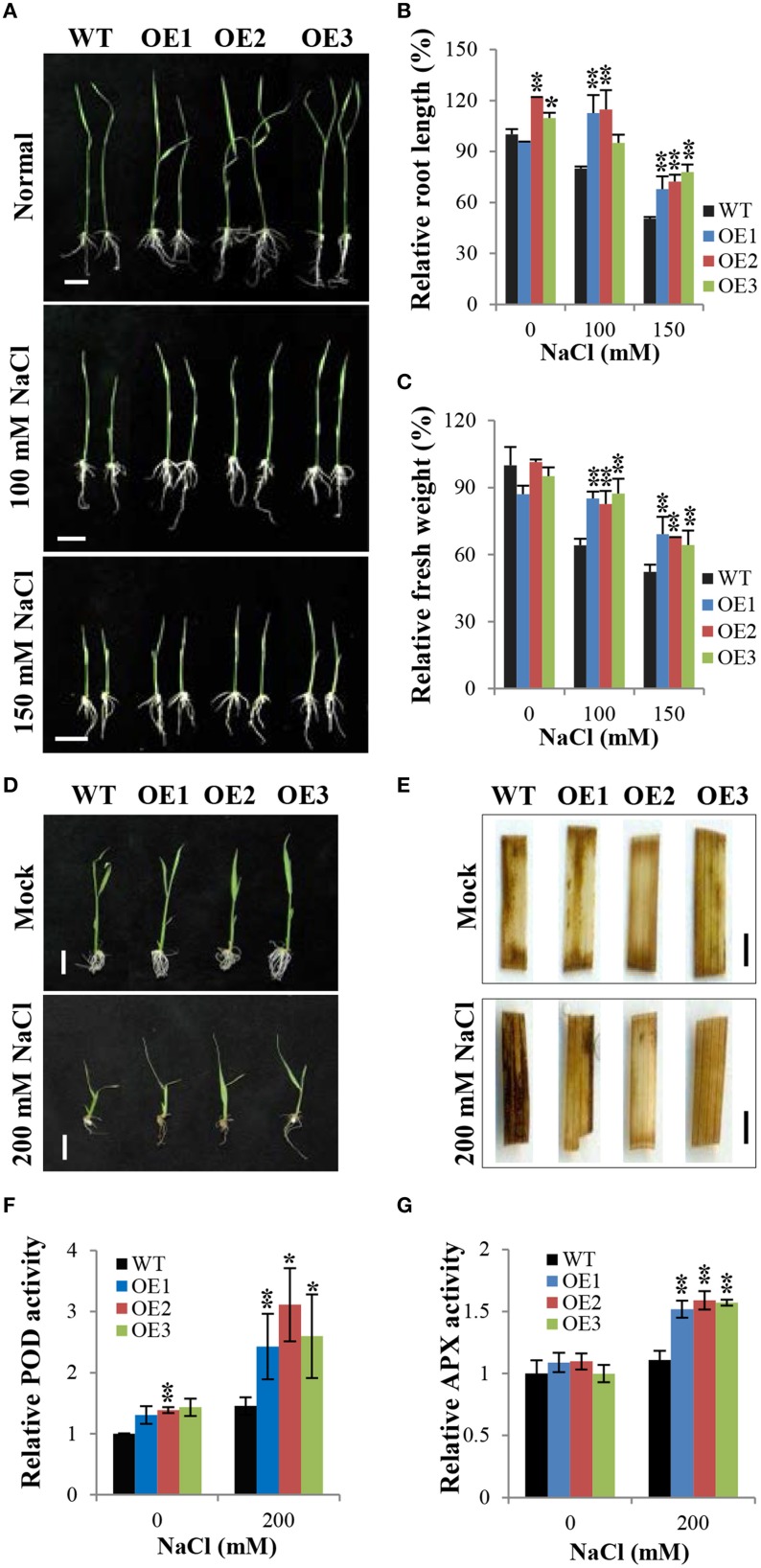
**Overexpression of ***OsCYP21-4*** increases salt stress tolerance in rice. (A,D)** Comparison of rice seedlings under NaCl stress. Transgenic plants (OE) and wild-type (WT) seeds were germinated on MS medium with or without hygromycin (50 mg/L), respectively, and after 3 days, they were transferred to fresh MS medium plates **(A)** or to distilled water containing 0, 150 or 200 mM NaCl **(D)** and grown for 5 days. **(B,C)** Relative root length and fresh weight of transgenic and WT control seedlings grown on MS medium plates. Error bars mean SE of three biological replicates. Asterisks indicate statistically significant differences between WT control and OsCYP21-4 OE transgenic plants (Student's *t*-test: ^*^p < 0.1, ^**^p < 0.01). **(E)** *In situ* H_2_O_2_ accumulation in plants shown in **(D)** was detected by 3,3′-diaminobenzidine (DAB) staining. The experiments were repeated twice with similar results obtained in each experiment. **(F,G)** Relative peroxidase and ascorbate peroxidase activities in WT and OsCYP21-4 OE plants. Error bars denote SE of three biological replicates. Asterisks indicate statistically significant difference between WT control and OsCYP21-4 OE transgenic plants (Student's *t*-test: ^*^p < 0.1, ^**^p < 0.05). Bars = 2 cm **(A,D)** and 2 mm **(E)**.

We generated OsCYP21-4-overexpressing transgenic plants containing the full-length ORF of *OsCYP21-4* under the control of the CaMV 35S promoter (for the constitutive expression) (Supplementary Figure 2A). We analyzed *OsCYP21-4* expression in WT and OsCYP21-4-overexpressing (OE: OE1, OE2, and OE3) transgenic plants via semi-quantitative RT-PCR. *OsCYP21-4* expression was higher in OsCYP21-4 OE plants than in WT (Supplementary Figure 2B). To investigate the phenotypes of OsCYP21-4 OE plants under abiotic stress conditions, we exposed 3-day-old WT and OsCYP21-4 OE seedlings to 1/2MS medium containing 0, 100, and 1500 mM NaCl for 5 days. As shown in Figure 5A, OsCYP21-4 OE transgenic plants were more tolerant to salt stress than WT plants. Although OsCYP21-4 OE2 and OE3 plants had slightly increased root lengths even under normal conditions, after 100 or 150 mM NaCl treatment, all OE plants had obviously different root lengths and fresh weights from those of WT plants. Under salt stress conditions, both the root lengths and fresh weights of the OE lines were approximately 10–20% higher than those of WT plants (Figures 5B,C).

Abiotic stresses such as salinity induce the accumulation of ROS, which are toxic molecules that induce oxidative injury in plants (Apel and Hirt, 2004). To determine whether OsCYP21-4 plays an important role in ROS homeostasis under salt stress, we investigated the accumulation of H_2_O_2_ by examining the precipitation of polymerized 3,3′-diaminobenzidine (DAB) in OsCYP21-4 OE and WT plants grown under high salinity conditions (Figures 5D,E). Three-day-old WT and OsCYP21-4 OE seedlings were treated with 200 mM NaCl in sterilized distilled water for 5 days. The salt tolerance phenotypes of OsCYP21-4 OE plants grown in salt solution were similar to those of OsCYP21-4 OE plants grown on the salt-containing 1/2MS medium (Figure 5D). Detached leaf fragments from salt stress-treated or untreated seedlings were incubated in DAB staining solution. An intense brown precipitate was observed in the leaves of WT plants stained with DAB after 5 days of exposure to high salinity. Under high salinity conditions, the intensity of DAB staining was markedly lower in the leaves of OsCYP21-4 OE plants than in the leaves of WT plants (Figure 5E). Under mock conditions, no difference was observed between DAB-stained leaf fragments of WT and OE plants. DAB *in vivo* staining showed that the salt stress tolerant OsCYP21-4 OE plants accumulated less H_2_O_2_ than the WT control plants. To investigate the possibility that the oxidative tolerance of the transgenic plants was associated with an increase in antioxidant enzyme activity, we conducted experiments to determine the activities of major H_2_O_2_ scavenging enzymes, POD, APX, and CAT, in transgenic plants. POD and APX activity levels were clearly higher in OsCYP21-4 OE than in WT plants (Figures 5F,G), whereas CAT activity was not shown any difference between OsCYP21-4 OE and WT plants (Supplementary Figure 3). The higher POD and APX enzyme activities of OsCYP21-4 OE plants under high salt stress conditions could lead to less H_2_O_2_ accumulation, and therefore to greater salt tolerance.

### *OsCYP21-4* OE plants are tolerant of H_2_O_2_ treatment and have increased peroxidase activity

**Figure 6:**
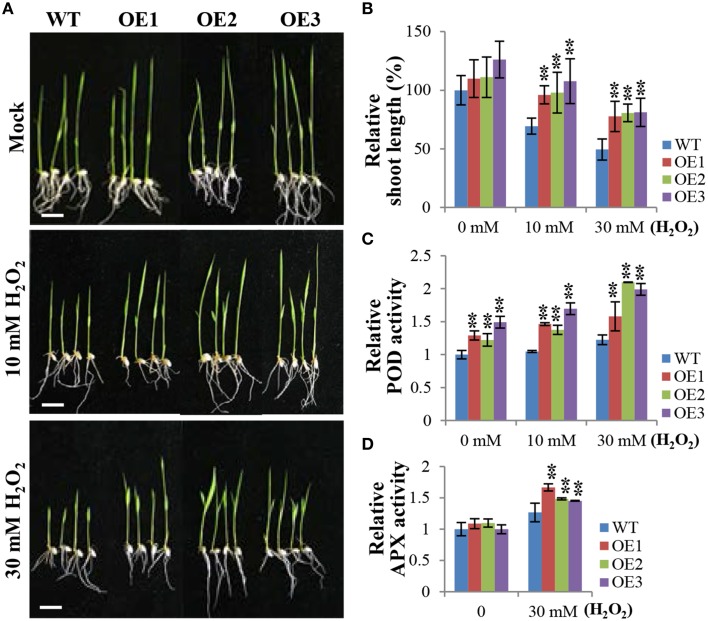
*****OsCYP21-4*** OE plants exhibit enhanced tolerance to H_2_O_2_ treatment and increased peroxidase activity. (A)** Comparison of rice seedlings under H_2_O_2_ treatment. Transgenic plants (OE) and wild-type (WT) seeds were germinated on MS medium with or without hygromycin (50 mg/L), respectively, and after 3 days, they were transferred to distilled water containing 10 mM or 30 mM H_2_O_2_ and grown for 5 days. **(B)** Relative shoot lengths of transgenic and WT control seedlings. **(C,D)** Relative peroxidase and ascorbate peroxidase activities in WT and OsCYP21-4 OE plants. Error bars represent SE of three biological replicates. Asterisks indicate significant differences between WT control and OsCYP21-4 OE transgenic plants (Student's *t*-test: ^**^p < 0.05). Bars = 2 cm.

As shown in Figure 5, OE plants exhibited salt tolerance and lower H_2_O_2_accumulation compared to WT plants. Therefore, we investigated whether exogenous treatment with H_2_O_2_ affects the growth of OsCYP21-4 OE and WT plants. Three-day-old WT and OsCYP21-4 OE seedlings were cultured in distilled water containing 0 (Mock), 10, and 30 mM H_2_O_2_ for 5 days. Under normal conditions (Figure 6A; Mock), there was no significant difference between WT and OsCYP21-4 OE plants. However, the overexpressing lines exhibited much better growth than WT seedlings under oxidative stress conditions (Figure 6A; 10 and 30 mM H_2_O_2_). To quantify the phenotypic differences under H_2_O_2_ stress conditions, we measured the fresh weights, root lengths, and shoot lengths of the plants, revealing significant increases in shoot lengths (Figure 6B) as well as fresh weights (data not shown) in all three OE lines. However, we did not detect clear differences in root length between WT and OE plants under H_2_O_2_ stress conditions (Figure 6A).

Like in the case of salt stress, we next conducted experiments to determine the activities of major H_2_O_2_ scavenging enzymes, POD, APX, and CAT, under H_2_O_2_ stress conditions. Even under normal growth conditions (Figure 6A; Mock), POD activity levels were 1.2–1.5-fold higher in the OsCYP21-4 OE plants than in the WT plants, suggesting that the increased *OsCYP21-4* expression of OE plants resulted in higher POD activity. Furthermore, the OE plants had 1.5–2-fold higher POD and APX activities than WT plants after H_2_O_2_ treatment (Figures 6C,D), whereas, there was no significant difference in CAT enzyme activity between OsCYP21-4 OE and WT plants under the same conditions (Supplementary Figure 3). These results suggest that the increased oxidative stress toleranace of the OsCYP21-4 OE plants may be due to their increased POD and APX activities.

Plants perceive and respond adaptively to abiotic stresses via pathways primarily controlled by the phytohormone, ABA. We therefore evaluated the response of OsCYP21-4 OE lines to ABA treatment. WT and OsCYP21-4 OE plants were grown on 1/2 medium supplemented with 5 μM ABA. The overexpressing lines exhibited much better growth than WT under ABA treatment conditions (Supplementary Figure 4A). We measured the fresh weights, root lengths, and shoot lengths of the plants, revealing noticeable differences in fresh weights in all three OE lines (Supplementary Figure 4B), as well as obvious differences in shoot and root lengths under ABA treatment conditions (data not shown). These results suggest that artificially up-regulating *OsCYP21-4* expression may improve the productivity of crops under environmental stress conditions, such as salt and oxidative stress.

## Discussion

Cyclophilins are ubiquitous proteins present in all organisms that are involved in a wide range of crucial cellular processes. Rice CYPs consist of 27 structurally distinct family members. Among these, OsCYP21-4 and OsCYP21-3 are putative mitochondria-localized cyclophilins, as previously revealed using prediction programs (Ahn et al., 2010). The current alignment results show that CYP21-4 homologs exist only in certain land plant species. Interestingly, *CYP21-4* is not present in photosynthetic cyanobacteria or Chlamydomonas, which supports the notion that *CYP21-4* genes originated after the divergence of Chlamydomonas from land plants (Figure 1). Sequence analyses also suggested that CYP21-4 homologs lack essential amino acid residues for PPIase activity/CsA binding. In practice, recombinant OsCYP21-4 protein exhibited nearly no PPIase activity in an *in vitro* assay (Figure 4). Not all cyclophilin proteins possess PPIase activity, indicating that their PPIase activity may have been lost during the course of evolution and gain of function independent of their PPIase activity (Kumari et al., 2013). Moreover, numerous studies revealed that multiple immunophilins have retained a chaperone function independent of their PPIase activity (Chakraborty et al., 2002; Mok et al., 2006). In agreement with the *in vitro* results, OsCYP21-4 might have a PPIase-independent chaperone-like function for preventing aggregation or maintaining the stability of the target protein in plant cells.

In the current study, the results of analysis using various programs to predict the subcellular localization of OsCYP21-4 (like AtCYP21-4) were conclusive, revealing its mitochondrial localization, although PSORT (employed before GolgiP) predicted that this protein is localized to the plasma membrane (Table 1). We co-expressed OsCYP21-4-GFP and the peroxisomal marker RFP-SKL, and probed the material with mitochondrial (MitoTrack) and ER (RFP-BiP) markers to confirm the localization of OsCYP21-4 *in vivo*. Contrary to expectations, OsCYP21-4 did not completely co-localize with MitoTracker, but the peroxisomal marker RFP-SKL was co-expressed with OsCYP21-4-GFP; in rare cases, the signals merged in particular regions. Furthermore, signals from the RFP-labeled ER marker, BiP (RFP-BiP) merged with those of OsCYP21-4-GFP in quite a few regions (Figure 2 and Supplementary Figures 1B,C). Meanwhile, GolgiP predicted that OsCYP21-4 is a Golgi-resident protein with a transmembrane domain. As a result, OsCYP21-4-GFP co-localized with cis α-GolgiManI-mCherry in most cases, depending on the transmembrane domain at the N-terminus (Table 1 and Supplementary Figure 1D). The fluorescence that did not co-localize with the α-ManI-mCherry signal may have resulted from trafficking of OsCYP21-4-GFP to another type of Golgi, such as the trans- Golgi network or post-Golgi compartments (Figure 2). The BFA treatment assay revealed that OsCYP21-4 localizes to the Golgi through ER transport. However, further experiments are needed to elucidate and verify the precise localization of OsCYP21-4 based on external stress and growth/developmental conditions.

Although an increasing body of evidence suggests that CYPs play an important role in diverse cellular processes, little is known about their physiological relevance and the molecular basis of their stress-responsive expression. *OsCYP21-4* is also responsive to multiple environmental stresses and to the representative stress-related phytohormone, ABA. Among the stresses examined, salt stress produced the strongest increase in *OsCYP21-4* expression, with transcript levels up to 10-fold higher than control levels, which suggests that OsCYP21-4 is involved in the salinity stress response (Figure 3). Soil salinity is a critical environmental constraint to crop production, and extensive research and biotechnological developments to facilitate the production of crops with improved salt tolerance are currently underway. OsCYP21-4 OE plants exhibited enhanced salinity tolerance and reduced H_2_O_2_ accumulation in response to treatment with high levels of salt (Figures 5A–E), indicating that OsCYP21-4 plays an important role in removing sources of ROS production in plant cells, which may explain why OsCYP21-4 OE seedlings exhibited significantly better growth than WT seedlings under high salinity conditions (Figure 5D). Glycans mature in the Golgi, and mutants defective in N-glycan modification are more salt-sensitive than WT plants (Kang et al., 2008). Therefore, it is also possible that the OsCYP21-4 OE plants are salt tolerant due to enhanced N-glycan maturation arising from *OsCYP21-4* overexpression.

As previous DAB staining results (Figure 5E) support the notion that OsCYP21-4 functions in ROS scavenging, we were interested in examining the behavior of OsCYP21-4 OE plants in response to direct treatment with H_2_O_2_. OsCYP21-4 OE plants exhibited less H_2_O_2_-induced damage than WT, suggesting that the transgenic plants were more tolerant to oxidative stress than WT plants (Figures 6A,B). To uncover the cause of this oxidative tolerance, we examined the activities of major H_2_O_2_ scavenging enzymes POD, APX and CAT under high salt and H_2_O_2_ treatment conditions. POD and APX enzyme activities were higher in the OsCYP21-4 OE plants than in the WT plants under both stress conditions (Figures 5F,G, 6C,D), suggesting that the transgenic plants possess a more efficient antioxidant network than the WT plants. This notion is corroborated by the accumulation of lower amounts of H_2_O_2_ in the transgenic plants. Therefore, the enhanced oxidative stress tolerance of the OsCYP21-4 OE plants may be attributed, at least in part, to their maintenance of low intracellular ROS pools, which is probably regulated by high peroxidase (POD and APX) activity. By contrast, CAT activity was not different between transgenic and WT plants (Supplementary Figure 3). Elucidating the relationship between peroxidase and CAT activities may provide important insights into the reciprocal physiological and biochemical effects of OsCYP21-4 under oxidative stress.

Plant CATs are important peroxisomal enzymes possessing a carboxyl terminal consensus sequence that primarily functions in peroxisomal import (Anderson et al., 1995; Mhamdi et al., 2012) and contributes to oxidative signaling. Plants possess large multigene families encoding secreted class III POD proteins (Passardi et al., 2004) and that play various physiological roles, such as the salt stress response, lignification, and defense against pathogens (Kawano, 2003; Passardi et al., 2005). Class III PODs are extensively glycosylated in the Golgi apparatus and are involved in the biosynthesis of lignin (Deepa and Arumughan, 2002). Therefore, we speculate that Golgi-resident OsCYP21-4 partly assist in the glycosylation of peroxidases, although the molecular mechanism underlying this process is currently unknown. The current results indicate that OsCYP21-4 confers oxidative stress resistance by regulating POD and APX enzyme activities in OsCYP21-4 OE plants. Further exploration of the enhanced stress tolerance of these plants may help shed light on the pivotal roles played by the Golgi under oxidative stress conditions.

## Author contributions

HC conceived and designed the study and wrote the manuscript, SL, HP, WJ, and AL performed the experiments and wrote the manuscript, DY, YY, HK, BK, and JA contributed research materials.

### Conflict of interest statement

The authors declare that the research was conducted in the absence of any commercial or financial relationships that could be construed as a potential conflict of interest.
