# Supplementary figures and images for: OsCYP21-4, a novel Golgi-resident cyclophilin, increases oxidative stress tolerance in rice
Source: Front Plant Sci. 2015 Oct 1;6:797. doi: 10.3389/fpls.2015.00797 (PMC4589654; doi:10.3389/fpls.2015.00797)

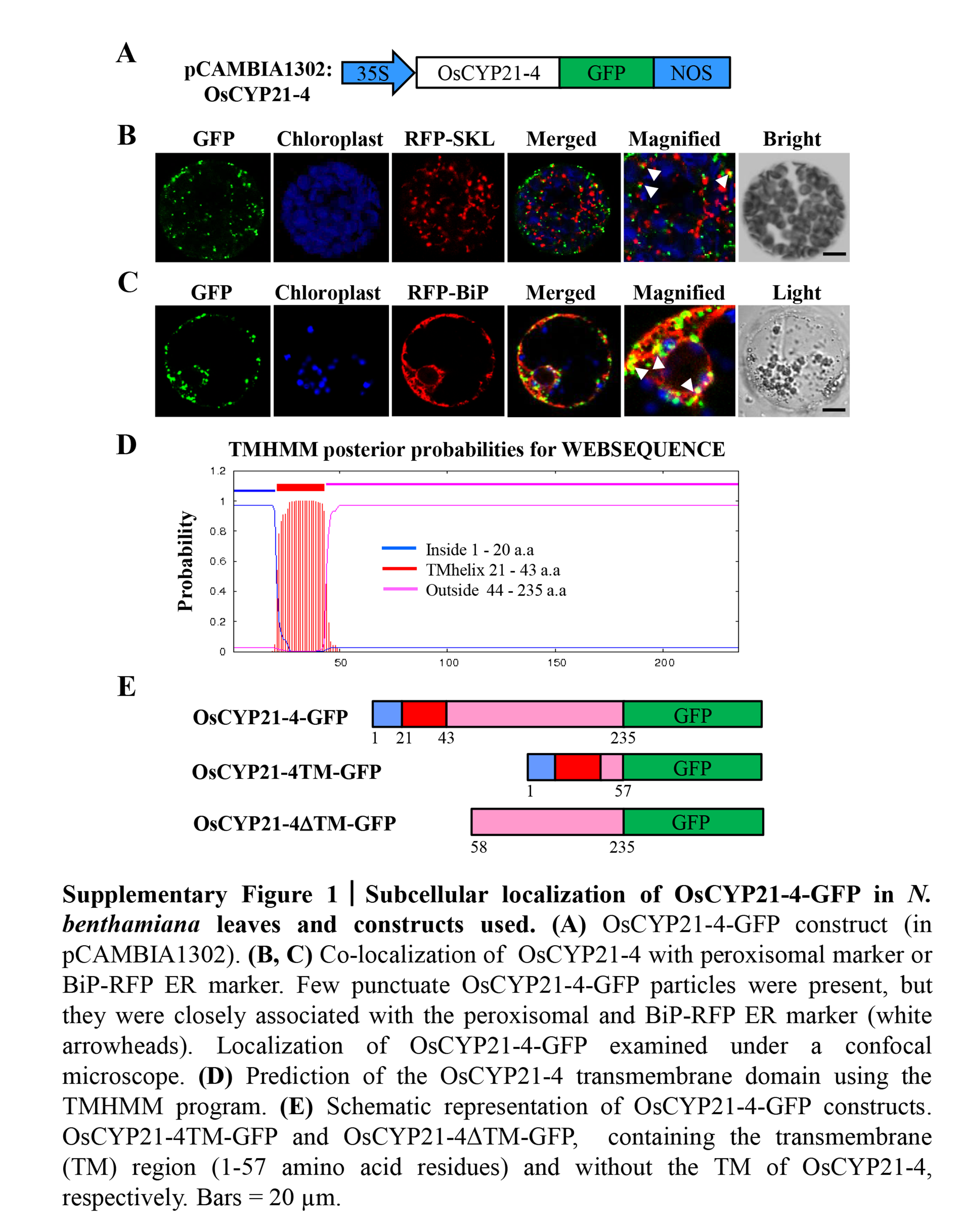

Supplement: Supplementary file 1 [file Image1.TIF]

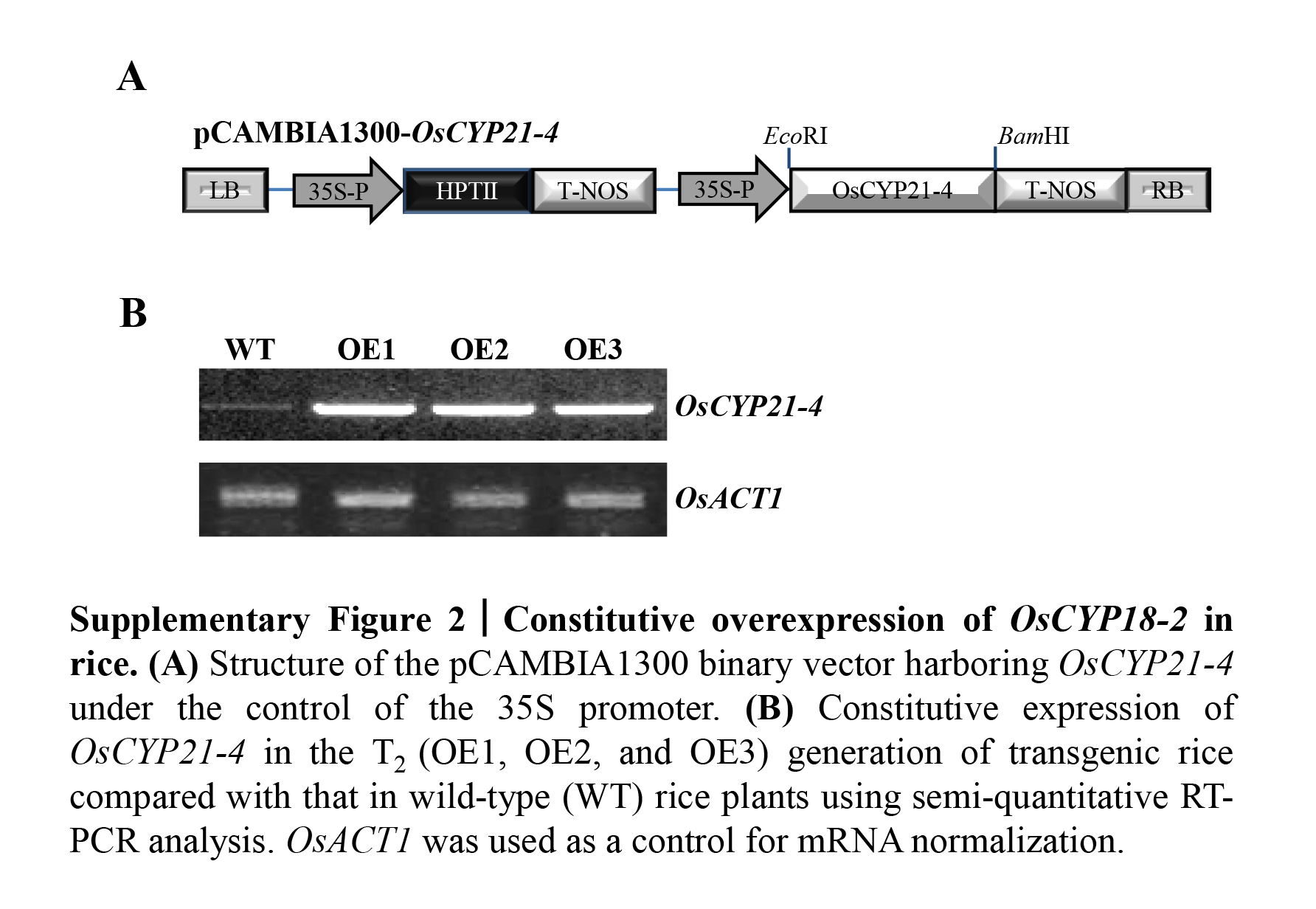

Supplement: Supplementary file 2 [file Image2.TIF]

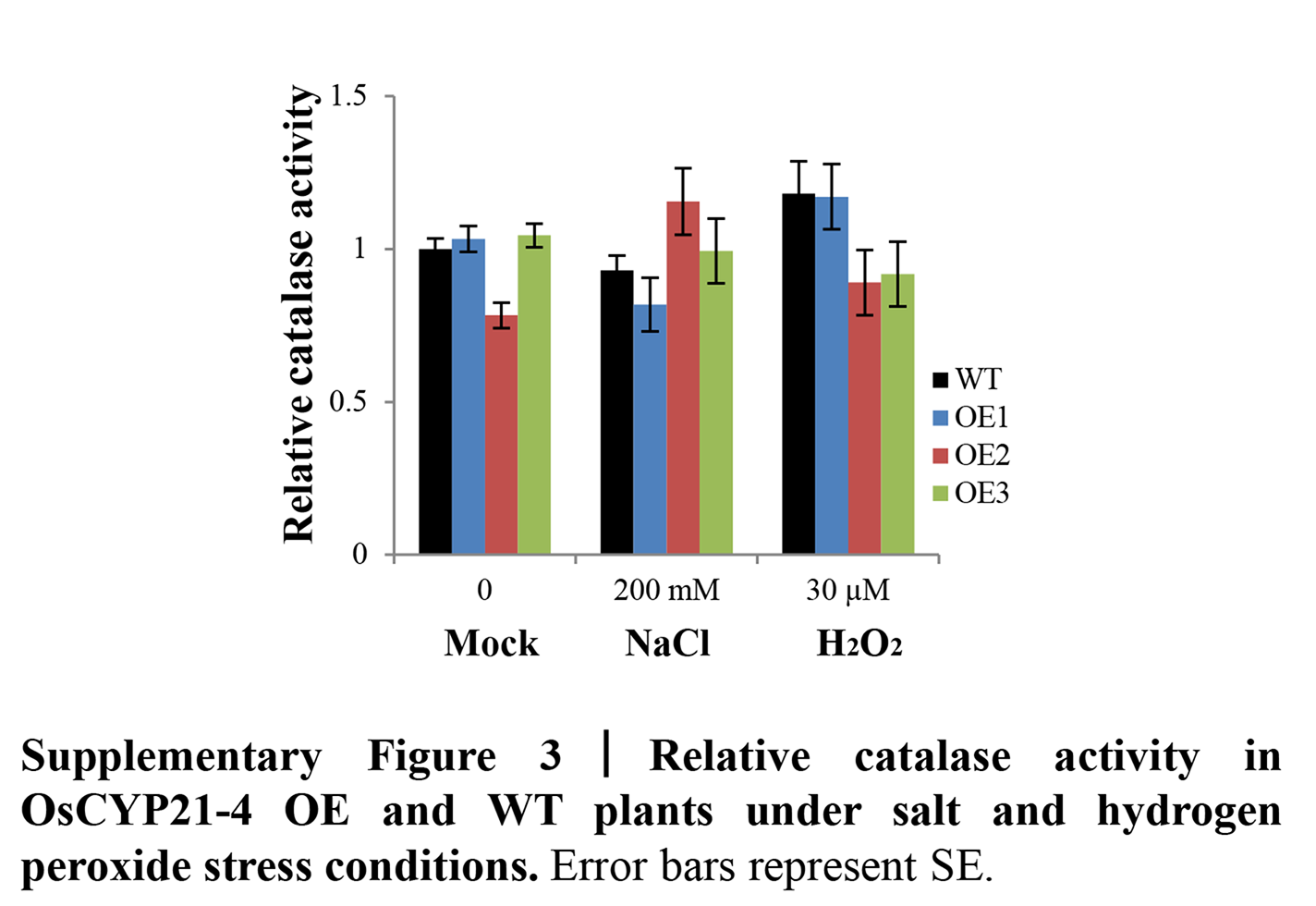

Supplement: Supplementary file 3 [file Image3.TIF]

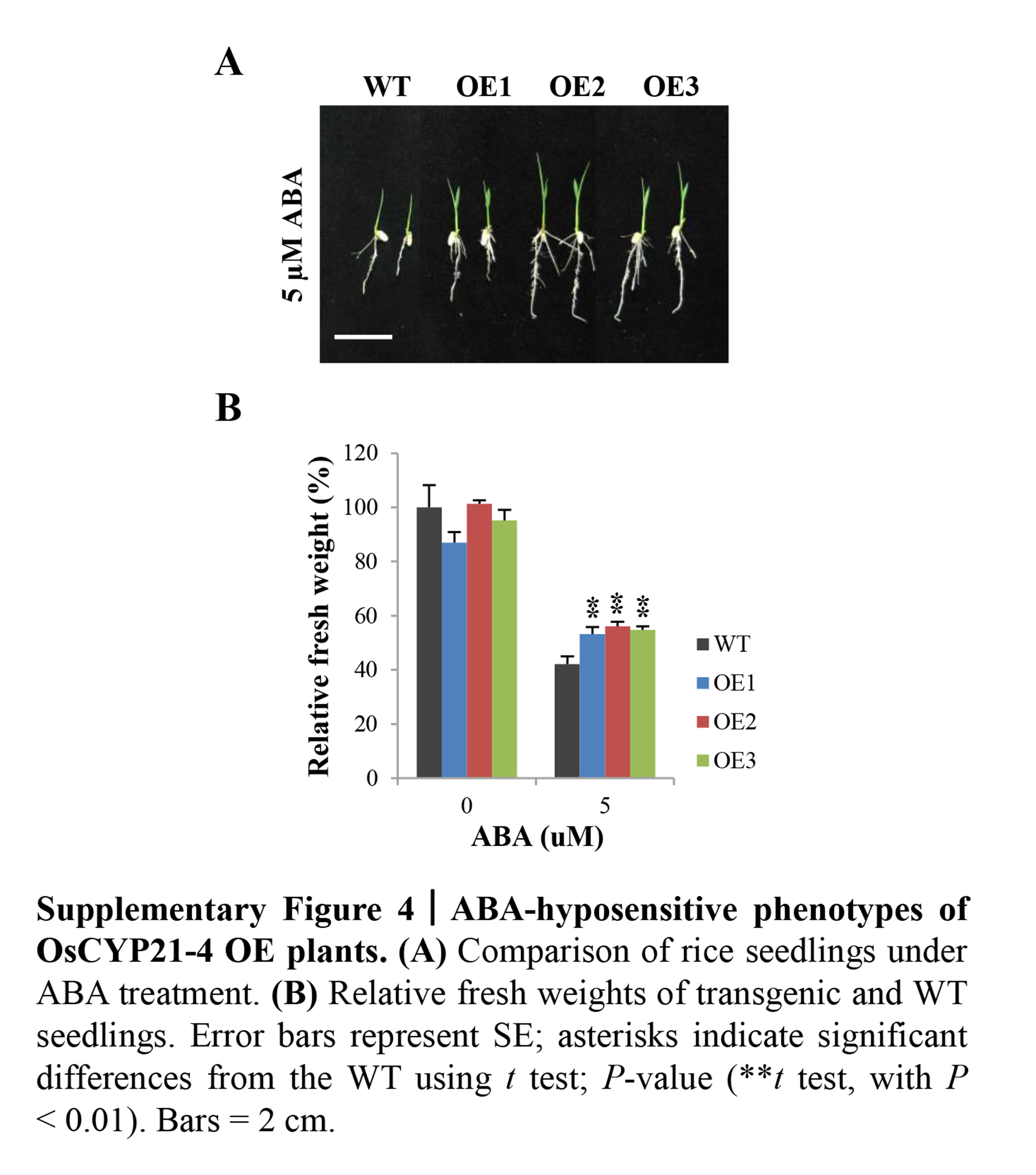

Supplement: Supplementary file 4 [file Image4.TIF]
