# Supplementary material for: OsCYP21-4, a novel Golgi-resident cyclophilin, increases oxidative stress tolerance in rice
Source: Front Plant Sci. 2015 Oct 1;6:797. doi: 10.3389/fpls.2015.00797 (PMC4589654; doi:10.3389/fpls.2015.00797)
Supplement: Supplementary file 5 [file Table1.PDF]

Supplementary Table 1. Gene-specific primers used in this study.

Gene name	Primer Sequences (F, forward; R, reverse[5’-3’])		Applications
OsCYP21-4	F: gaattcatggcgaggataaagccgaagcaattg	R: ggatcctcagctcaaagcttgctgttttagcgtgatg	Transgenic plant (pCAMBIA1300 cloning)
OsCYP21-4	F: cagccatggcaacgacgataaagccgaagc	R: cagccatgggctcaaagcttgctgttttagcg	Subcellular localization (pCAMBIA1302 cloning)
OsCYP21-4TM	F: tacccatggcgaggataaagccgaagc	R: aatccatggcttcagtctcgagcattggcc	Subcellular localization (pCAMBIA1302 cloning)
OsCYP21-4ΔTM	F: tatccatggatctccctcgtgctgagca	R: aatccatggcgctcaaagcttgctgttttagcg	Subcellular localization (pCAMBIA1302 cloning)
OsCYP21-4	F: taccatatggcgaggataaagccgaagcaa	R: atgcggccgcgctcaaagcttgctgttttagcgt	Protein expression (pET41b cloning)
OsCYP21-4	F: atggcgaggataaagccgaagcaattg	R: tcagctcaaagcttgctgttttagcgtgatg	RT-PCR or qRT-PCR
OsACT1	F: catgctatccctcgtctcgacct	R: cgcacttcatgatggagttgtat	RT-PCR or qRT-PCR
